# Comparison of ISS, NISS, and RTS score as predictor of mortality in pediatric fall

**DOI:** 10.1186/s41038-017-0087-7

**Published:** 2017-08-08

**Authors:** Kapil Dev Soni, Santosh Mahindrakar, Amit Gupta, Subodh Kumar, Sushma Sagar, Ashish Jhakal

**Affiliations:** Department of Trauma Surgery and Critical Care, Jai Prakash Narayan Trauma Centre, AIIMS, New Delhi, India

**Keywords:** Pediatric fall, Trauma score system, ISS, NISS, RTS

## Abstract

**Background:**

Studies to identify an ideal trauma score tool representing prediction of outcomes of the pediatric fall patient remains elusive. Our study was undertaken to identify better predictor of mortality in the pediatric fall patients.

**Methods:**

Data was retrieved from prospectively maintained trauma registry project at level 1 trauma center developed as part of Multicentric Project—Towards Improving Trauma Care Outcomes (TITCO) in India. Single center data retrieved from a prospectively maintained trauma registry at a level 1 trauma center, New Delhi, for a period ranging from 1 October 2013 to 17 February 2015 was evaluated. Standard anatomic scores Injury Severity Score (ISS) and New Injury Severity Score (NISS) were compared with physiologic score Revised Trauma Score (RTS) using receiver operating curve (ROC).

**Results:**

Heart rate and RTS had a statistical difference among the survivors to nonsurvivors. ISS, NISS, and RTS were having 50, 50, and 86% of area under the curve on ROCs, and RTS was statistically significant among them.

**Conclusions:**

Physiologically based trauma score systems (RTS) are much better predictors of inhospital mortality in comparison to anatomical based scoring systems (ISS and NISS) for unintentional pediatric falls.

## Background

Unintentional injuries were responsible for 3.9 million deaths and over 138 million disability adjusted life-years in 2004, and among them, 90% are from low- and middle-income countries (LMIC) [1–5]. A global pilot study on unintentional injury among childhood in the developing countries reveals that more than half had a history of falls (52%) [6, 7]. Principle of trauma care is same for the adult and pediatric, but the anatomical and physiological status of children increases the risk of severe injury with minor fall [8–10]. A systematic review on risk of pediatric (0–6 years) fall reveals that age, sex, and poverty are independent risk factors for injuries [11].

Trauma scoring system is an essential component of triage, to compare the different models of trauma care and its quality [12]. Previous study showed Glasgow Coma Scale (GCS) as a superior predictor for inhospital mortality in comparison to Pediatric Trauma Score (PTS) [13, 14] and Injury Severity Score (ISS) raising concerns about the suitability of using anatomical based scoring systems. Potoka et al. developed novel scoring system based upon age-specific physiological criteria and concluded physiological-based score as a tool for prediction of survival [15]. Previous attempts to compare and validate different scoring systems in pediatric patients have yielded varying results [15–17]. However, an ideal tool for prediction in pediatric trauma remains elusive. The performance of trauma score may vary on the different systems of care as well as different mechanism of injury.

Henceforth, the aim of the study is to compare three different severity score ISS, New Injury Severity Score ﻿(NISS), Revised Trauma Score (RTS) systems sensitivity among the pediatric patients arriving at emergency department of the tertiary level 1 trauma center after fall.

## Methods

The data was collected from the trauma registry developed under Towards Improving Trauma Care Outcome in India (TITCO), and the ethical permission was availed from the Institute Ethical Committee (All India Institute of Medical Sciences) for the study. The present paper is a secondary analysis of a single center data from a multicentre study (no. IEC/NP-279/2013 RP-01/2013).

Data was retrieved from a prospectively maintained trauma registry at a level 1 trauma center, New Delhi, for a period ranging from 1 October 2013 to 17 February 2015. The registry includes all the patients admitted and died in the emergency room (ER). Patients were further screened; all pediatric fall patients were identified in the dataset and included for analysis. Patients with single long-bone injuries and brought dead were excluded.

The ISS, NISS, and RTS were calculated retrospectively based on internationally agreed definitions on Abbreviated Injury Score (AIS) coding, calculated by certified coders (the Association for Advancement of Automotive Medicine (AAAM)). All the injuries of each patient are coded as per the AIS. There are nine body regions in AIS. A composite score is formed by combination of these nine into six, and from these six regions, the highest value from three regions are selected. These highest values are squared and added together to calculate to be called as ISS. The highest three scoring from any of the nine AIS region is squared and added to achieve a composite score called as NISS. RTS is calculated as per the formula: RTS = 0.9368GCS + 0.7326SBP + 0.2908RR.

The data were further analyzed using SPSS 23.

### Statistical analysis

Chi square test and independent *t* test were applied for categorical and continuous variable hypothesis testing. Receiver operating curve (ROC) was used to predict the mortality with three different trauma score systems. The variables needed to compute RTS, ISS, and NISS were available and used from dataset; however, PTS variables were unavailable.

## Results

**Fig. 1:**
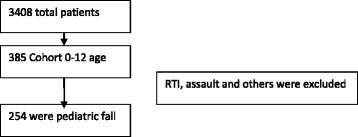
Flow chart

Out of 3408 patients screened, 385 were in pediatric age group (0–12 years). Two hundred fifty-four pediatric patients were brought with history of fall and rest with other mechanism of injury (Fig 1). Falls were analyzed further, 68.9% were male, and among the mode of transport, taxi (31.1%), private car (34.3%), and ambulance (32.7%) were equally preferred. Nine 3.5% patients died early in the course within the ER with overall inhospital mortality of 15% in the pediatric fall cohort. One third patients presented with low GCS (less than 8) and were intubated. One third (33.5%) of the fall cases occurred between 13﻿ and 16 p.m. and most (64.5%) within 8 h of the day (13–20 p.m.). More than one third (37%) of patients w﻿ere in toddler (2–3 years) with few in preschooler (4–6 years) (27.2%) and school age (7–12 years) (24.4%) (Table 1). However, only few (17.7%) of them were having systolic blood pressure (SBP) less than or equal to 90 mmHg. Mean age of the patients was 4.5 ± 3 years, and mean of (SBP), SpO_2_, respiration rate, heart rate, and GCS were 105.25 ± 23.3 mmHg, 93.29 ± 17.6%, 19.4 ± 4.9 breaths/min, 106.9 ± 28.2 beats/min, and 11.24 ± 4.3 respectively. Mean ISS, NISS, and RTS scores were 11.87 ± 7.6, 17.15 ± 10.1, and 6.71 ± 1.6 respectively (Table 2).

**Table 1:** Descriptive analysis of categorical variable *N* = 254

Variable	Frequency	Percentage
Male	175	68.9
Admitted	245	96.5
ED death	9	3.5
Mode of transport		
Taxi, motor rickshaw	79	31.1
Private car	87	34.3
Ambulance	83	32.7
Police	1	0.4
Other	4	1.6
SBP on arrival		
≤90 mmHg	45	17.7
>90 mmHg	209	82.3
GCS on arrival		
Severe (≤8)	78	30.7
Moderate (9–12)	42	16.5
Mild (13–15)	134	52.8
Intubation within an hour		
Yes	90	35.4
Intubated before arrival	13	5.1
ICD within an hour	14	5.5
Death	38	15.0
Age in years		
0–1	29	11.4
2–3	94	37.0
4–6	69	27.2
7–12	62	24.4
Time of incident (in hours) (*N* = 245)		
1–4 a.m.	3	1.2
5–8 a.m.	9	3.7
9–12 a.m.	61	24.9
13–16 p.m.	82	33.5
17–20​ p.m.	76	31.0
21–24​ p.m.	14	5.7

*ED* emergency department, *SBP* systolic blood pressure, *GCS* Glasgow Coma Scale

**Table 2:** Descriptive analysis of continuous variables

	Total	Range	Mean	Std. deviation
Age (years)	254	0–12	4.50	3.0
SBP (mmHg) (on arrival to ED	253	0–190	105.25	23.3
Heart rate (beats/min) (on arrival to ED)	254	0–256	106.90	28.2
GCS (on arrival to ED)	254	3–15	11.24	4.3
SpO_2_ (on arrival to ED)	254	0–100	93.29	17.6
Respiration rate (breaths/min) (on arrival to ED	250	0–40	19.40	4.9
ISS	230	1–42	11.87	7.6
NISS	230	1-50	17.15	10.1
RTS	250	0.29–7.84	6.71	1.6

*SBP* systolic blood pressure, *ED* emergency department, *GCS* Glasgow Coma Scale, *ISS* Injury Severity Score, *NISS* New Injury Severity Score, *RTS* Revised Trauma Score

Percentage of patients with SBP less than or equal to 90 mmHg and severe GCS on arrival were statistically high among nonsurvivors. The mean ISS, RTS, and respiratory rate per minute were low among the nonsurvivors than survivors whereas heart rate per minute and NISS among the nonsurvivors were higher. Among these vital signs and severity scores and RTS had a statistical difference among the survivors to nonsurvivors (Table 3).

**Table 3:** Comparison selected variables among survivors and nonsurvivors

Variable		Survivors	Nonsurvivors	*P* value
Sex (*N*, %)	Male	148, 68.5	27, 71.1	0.757
	Female	68, 31.5	11, 28.9	
Referral (*N*, %)	Direct	101, 46.8	18, 47.4	0.853
	Referred	115, 53.2	20, 52.6	
Mode of transport (*N*, %)	Taxi, motor rickshaw	69, 31.9	10, 26.3	0.295
	Private car	75, 34.7	12, 31.6	
	Ambulance	68, 31.5	15, 39.5	
	Police	1, 0.5	0, 0	
	Other	3, 1.4	01, 2.6	
SBP (*N*, %)	≤ 90 mmHg	27, 12.5	18, 47.4	<0.001
	>90 mmHg	189, 87.5	20, 52.6	
GCS (*N*, %)	Severe	47, 21.8	31, 81.6	<0.001
	Moderate	40, 18.5	2, 5.3	
	Mild	129, 59.7	5, 13.2	
ISS (Mean ± SD)		11.87 ± 7.61	11.68 ± 7.40	0.877
NISS (Mean ± SD)		17.08 ± 9.89	17.52 ± 11.36	0.818
RTS (Mean ± SD)		7.1308 ± 0.99	4.3946 ± 2.20	<0.001
Respiratory rate (Mean ± SD)		19.06 ± 4.00	18.16 ± 8.22	0.094

*SBP* systolic blood pressure, *GCS* Glasgow Coma Scale, *ISS​* Injury Severity Score, *NISS* New Injury Severity Score, *RTS* Revised Trauma Score

**Fig. 2:**
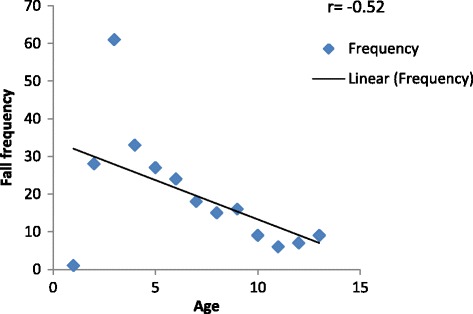
Scatter diagram representing the age and frequency of the fall among pediatric patients

**Fig. 3:**
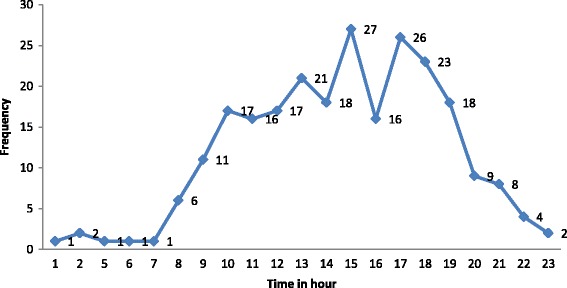
Line graph presenting the number of frequency and time of arrival to the hospital (in hours)

Negative linear correlation (*r* = −0.52) was found between the age and frequency of fall signifying decrease in incidence of fall as the age progresses (Fig. 2). Figure 3 represent corelation between arrival time to the ER and number of pediatric patients with fall .

ISS, NISS, and RTS were calculated by the AAAM coders and were assessed to predict the sensitivity of these tools among the pediatric fall cases. Receiver operating characteristic curve was used to compare the sensitivity of three different trauma scores.

**Fig. 4:**
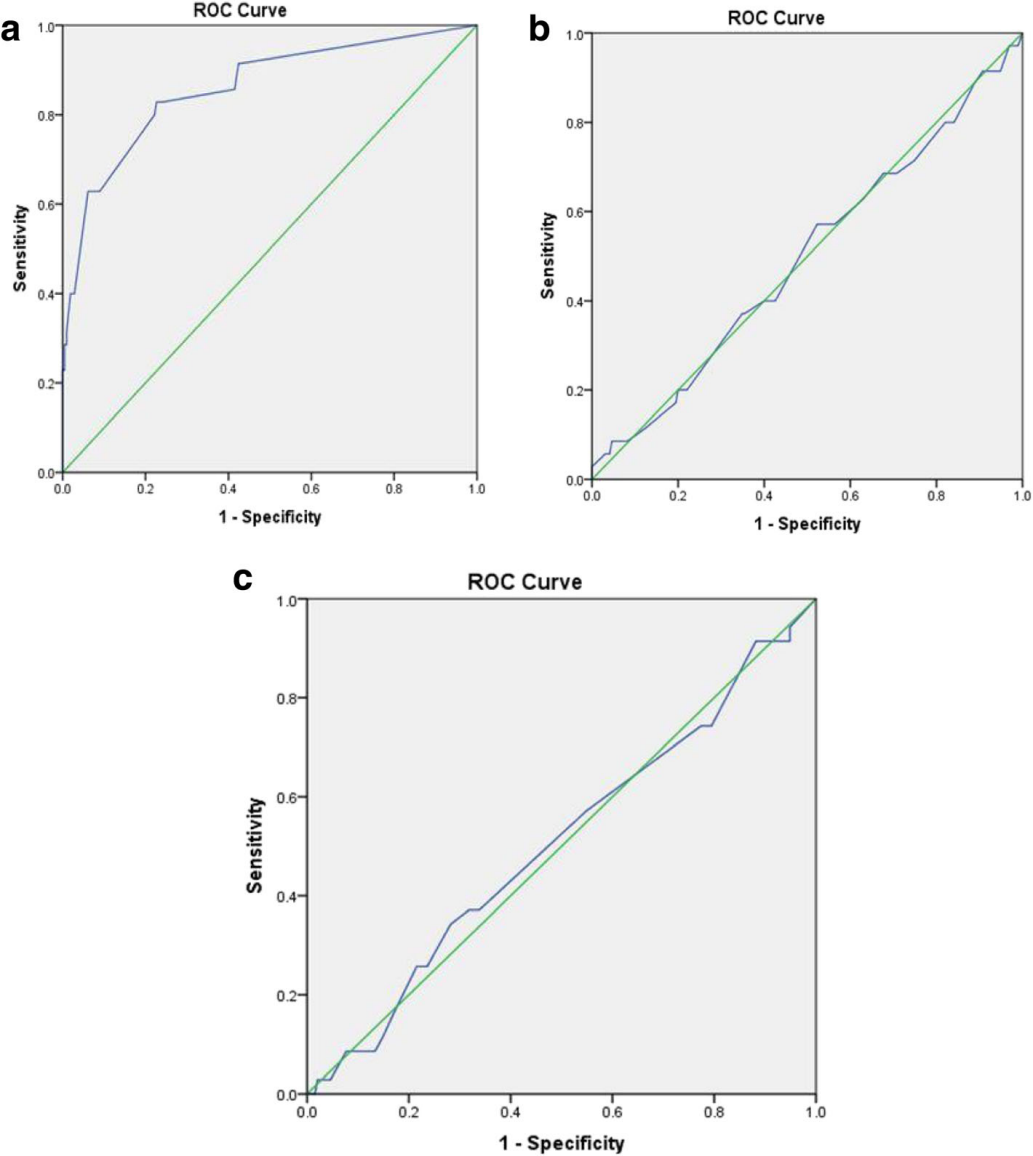
Receiver operating curve (ROC) curve of **a** Revised Trauma Score (RTS), **b** Injury Severity Score (ISS), and **c** New Injury Severity Score (NISS)

Among these three trauma scores, ISS, NISS, and RTS were having 50, 50, and 86% of area under the curve (Fig. 4). RTS was statistically significant among them. Performance of ISS and NISS was considerably low (Table 4).

**Table 4:** Area under the curve for different severity scores

Score	Area under curve	*P* value
ISS	0.507	0.901
NISS	0.495	0.959
RTS	0.860	<0.001

## Discussion

We compared the three standard trauma scores, i.e., RTS, ISS, NISS, in the pediatric fall population and found that RTS was most sensitive to predict the inhospital mortality. This may be attributed to higher propensity of respiratory and neurological derangements seen frequently in pediatric patients in absence of anatomical injuries. It may be speculated that since ISS depends on anatomical injuries, a conservative approach for radiological investigations may miss substantial number of injuries and could lead to poor performance of ISS and NISS in the given cohort of pediatric fall. Previous studies has shown that vital signs like heart rate, respiratory rate, and GCS may be more accurate predictors for inhospital mortality than ISS and NISS and may depend on age [15, 18]. Similarly in adults, a multicentric study in India concluded that RTS is a better predictor of inpatient mortality than ISS and NISS [12]. However, performance of RTS in particular cohort of pediatric fall was lacking. The present study demonstrates the consistent superiority of RTS against ISS and NISS too. Among children, a smaller injury or fall may result in severe derangements and have adverse outcome due to their physiological vulnerability, immature immune system, and fragile structure [8]. El-Gamasy et al. 2016 studied Pediatric Trauma Big Score as a predictive for mortality in pediatric polytraumatized patients and concluded that it has higher sensitivity and specificity than other trauma scores (PTS and NISS) [19, 20].

### Limitation

This study is limited to the patients admitted in one of the tertiary trauma level 1 trauma center in Delhi. It is a secondary analysis of prospectively collected dataset; hence, pediatric trauma score could not be compared to RTS, ISS, and NISS since the variables needed to compute were not available. Therefore, the question whether PTS or RTS is a better predictor for inhospital mortality in pediatric fall cohort could not be answered in the present study. The study could not calculate Pediatric Big Score due to lack of required variable in the data base.

## Conclusions

To conclude, physiologically based trauma score systems (RTS) are much better predictors of inhospital mortality in comparison to anatomical based scoring systems (ISS, NISS) for unintentional pediatric falls. Fall constitutes major mechanism of unintentional injury to pediatric cohort. An identification of more precise tool may help clinician to better identify the patients at risk of worse outcome at an early stages and could lead to institutions of prophylactic measures.
